# A generalizable and tunable engineered ecosystem provides a clear route to prosperity and well-being to harness the world’s aquatic “blue” food systems to help end hunger: A perspective

**DOI:** 10.3389/frfst.2022.886808

**Published:** 2022-09-21

**Authors:** Shengwen Calvin Li, Jian-Guo He

**Affiliations:** 1 University of CA-Irvine School of Medicine, Children’s Hospital of Orange County, Orange, CA, United States; 2 State Key Laboratory for Biocontrol, School of Marine Sciences, Sun Yat-sen University, Zhuhai, China; 3 School of Life Sciences, Sun Yat-sen University, Guangzhou, China

**Keywords:** shrimp, AQUAL, aquaculture, viral control, predator, biological control

## Abstract

Seafood security is essential in modern society. In 2013, Bush and colleagues stated, ‘Aquaculture, farming aquatic organisms, provides close to 50% of the world’s supply of seafood, with a value of United States $125 billion. It makes up 13% of the world’s animal-source protein (excluding eggs and dairy) and employs an estimated 24 million people’. With the increase in the human population and reducing fishing resources, humans increasingly rely on aquacultural products as the primary protein sources for many countries. Aquacultural productivity has been improving in recent years, and in certain countries, the aquaculture output is more than the fishing output. For example, Chinese aquaculture production is more than fishing output, which provides one-third of animal protein. Thus, intensive aquaculture has become the main supply with global aquatic products (FAO). In recent years, it is estimated that each person consumption of aquaculture products is 130 kg in some countries (Iceland). Here, we illustrate the road blocker in farmed shrimp production and provide our resolution. The global pandemic of white spot syndrome (WSS), caused by the white spot syndrome virus (WSSV), bears a devastating economic loss in farmed shrimp production, thereby jeopardizing seafood security. Currently, there is no effective control for WSS. Conventional single-species intensive farming removes the spatiotemporal interaction between different species. We hypothesize that establishing the spatiotemporal interface of a predator–prey may control WSS outbreak. We search for the pathways for the mechanisms by which predator–prey species interact and compete across spatial scales to characterize WSSV dispersal at regional scales for the local spatiotemporal structure of viral transmission. Thus, we create a generalizable and tunable engineered ecosystem that provides a clear route to prosperity and well-being to harness the world’s aquatic “blue” food systems to help end hunger.

## 1 Introduction: Current trend worldwide of ending hunger using the biodiversity of nature–the mission, the blue food

The System of Environmental–Economic Accounting gross ecosystem product (GEP) includes climate change and the derived effectors. “The United Nations leaders envisioned that climate change might alter the human–environment interface that influences biodiversity sustainability, which warrants a clear route to prosperity and well-being worldwide 50 years ago” (Nature-Editorial, 2022). “However, the world remains in a crisis of the earth’s limited supply of natural resources (diminishing food and energy resorts) due to accelerated climate and biodiversity exploiting—the warnings derived from hunger, wars, environmental degradation, and natural-resource depletion now hit even closer, threatening the survival of mankind.”

“A quarter of a century ago, Costanza et al.1 put forward an estimate for the economic value of global ecosystem services—the benefits people obtain from ecosystems. The authors valued these at United States $33 trillion per year” (Daily and Ruckelshaus, 2022). “A drive to integrate ecology and economics was under way and through it came detailed recognition of societal dependence on nature. The arc of this work grew with the 1995 Global Biodiversity Assessment5 and the 2001 launch of the Millennium Ecosystem Assessment6, galvanizing researchers globally to assess the status and trends in ecosystems and their services to society as a foundation for policymaking.” “Also under way is systems change: a transformation of mindsets and institutions—their policies, practices, and norms—to address causes rather than symptoms,” which together contribute approximately $222 billion annually in development-aid financing (see go.nature.com/3x0i56q), committed to mainstreaming nature into our policies, analysis, assessments, advice, investments, and operations by 2025 (see go.nature.com/3aobzdz). “The System of Environmental–Economic Accounting” (see https://seea.un.org and go.nature.com/38lc38h) and a new metric, derived from this accounting, was called “gross ecosystem product (GEP).” “These capabilities hold great potential for further advances in policy, planning, finance, and operations.” More than three billion people rely on the ocean to make a living, most of whom are in developing countries. For some 17% of the world’s population, fisheries and aquaculture provide the main source of animal protein. For the least-developed countries, fish contributes about 29% of animal protein intake; in other developing countries, it accounts for 19%.

“As the global population increases, the demand for seafood is expected to rise, too.” Already, Africa and Asia have doubled fish production over the past few decades. Globally, fish consumption is set to rise by approximately 15% by 2030 (Bush et al., 2013, Science, 341, 1067–1068). Each person is expected to have 130 kg (Naylor et al., 2000, Nature, 405, 1017–1024).

Although ocean ecosystems are strained by climate change, overfishing, and more, studies nevertheless suggest that seafood can be expanded sustainably to meet future food demands3. “Last year, international efforts promoting this approach included the Blue Food Assessment (a joint initiative of 25 research institutions) and the United Nations Food Systems Summit” (Hendriks, 2022).

Nature’s Editorial, 15 September 2021, harness the world’s aquatic “blue” food systems to help end hunger Harness the world’s aquatic ‘blue’ food systems to help end hunger (nature.com) (doi: https://doi.org/10.1038/d41586-021-02476-9) has launched “journals in the Nature Portfolio that shine a spotlight on how aquatic food systems—sometimes called blue foods—can help to end hunger and accelerate the creation of a truly sustainable global food system” (see go.nature.com/3nw8qbf). The research is part of the Blue Food Assessment, a collaboration involving more than 100 researchers. It is the first systematic assessment of how aquatic food contributes to food security, helping to build a fuller picture of the global food system beyond food from agriculture. Of these articles, Lotte Lauritzen on “A spotlight on seafood for global human nutrition” https://www.nature.com/articles/d41586-021-02436-3; and Christopher D. Golden, J. Zachary Koehn, and Shakuntala H. Thilsted on “Aquatic foods to nourish nations” and “Aquatic foods have been neglected by researchers and policymakers are recognizable https://www.nature.com/collections/fijabaiach—all caught our attention. In particular, we marvel at the advocates on, “Ending Hunger: Science must stop neglecting smallholder farmers” (Editorial 12 October 2020) https://www.nature.com/articles/d41586-020-02849-6, which states, “Policymakers urgently need ideas on ways to end hunger. But a global review of the literature finds that most researchers have had the wrong priorities to make a difference to the lives of the 690 million people who go hungry every day.” The team was able to identify ten practical interventions that can help donors to tackle hunger, of which the World Food Programme is the United Nations’ primary agency in the effort to eliminate hunger, which includes the Sustainable Development Goal (SDG) to end hunger by 2030.”

Climate changes evolve so that “the species richness of many taxa is higher near the equator, and ecologists have long hypothesized that this pattern is linked to stronger interactions between species (e.g., competition and predation) in the tropics.” However, empirical evidence shows that the strength of species interactions varying with the latitude is limited. Ashton et al. (2022) tested whether predation on benthic marine communities is higher at lower latitudes. Using a standardized experiment at 36 sites along the Pacific and Atlantic coasts of North and South America, the authors found both greater predation intensity (consumption rate) and stronger impact on benthic communities nearer the equator. These trends were more strongly related to water temperature than to latitude, suggesting that climate warming may influence top–down control of communities. “Aquatic foods such as fish, shellfish, and seaweed, collectively known as blue food, show potential for reducing some adverse environmental effects of global food production. In this week’s issue, Gephart et al. (2021) provided standardized estimates for a range of environmental pressures for diverse blue foods, representing about three-quarters of global production. The researchers looked at greenhouse-gas emissions, nitrogen and phosphorus pollution, and freshwater and land use. They found that farmed bivalves and seaweed generate the lowest emissions and use the fewest land and water resources. They identified many finfish options with low emissions and resource use, both farmed and wild-caught.”

“But a burning question remained largely unanswered: how to move from knowledge to action.” We recently published a convenient polyculture system that controls a shrimp viral disease with a high transmission rate (Wang et al., 2021), which aligns well with the current trend worldwide of ending hunger using the biodiversity of nature—Collaborative problem-solving and an integrated food system can deliver seafood protein, sustainably, to a world that increasingly needs it (Hendriks, 2022).

## 2 An example: The issue, the solution, and the achievement

All of the aforementioned visionary articles pointed out that researchers and policymakers have neglected aquatic foods; it is time to recognize them. However, a lack of specific action plans surfaced on small-scale farmers, especially those from low-income countries. For example, we reported that white spot syndrome (WSS), which is caused by the WSS virus (WSSV), leads to catastrophic economic losses for the global shrimp aquaculture industry of over $1 billion annually, outweighing the losses due to other major crustacean diseases. WSS pandemics primarily occur with the sequential transmission of WSSV from healthy shrimp that consume dead WSSV-infected shrimp to other healthy shrimp. Because of the high efficiency and low negative environmental impact, culturing specific pathogen-free (SPF) shrimp is the most widely used strategy for controlling WSS outbreaks. Disease prevention using SPF shrimp is only likely to succeed if accompanied by stringent and sophisticated pathogen-exclusion management practices. However, small-scale farmers, especially those from low-income countries, have limited access to or cannot afford SPF broodstock.

Moreover, they do not have the infrastructure and technical skills to apply the required biosecurity practices for culturing SPF shrimp. Therefore, these limited resource farms, which cultivate shrimp to improve livelihoods, are more vulnerable to WSS outbreaks than industrial farms. Most of these small farms have suffered from financial collapse due to production losses caused by WSS outbreaks (Wang et al., 2021).

**FIGURE 1:**
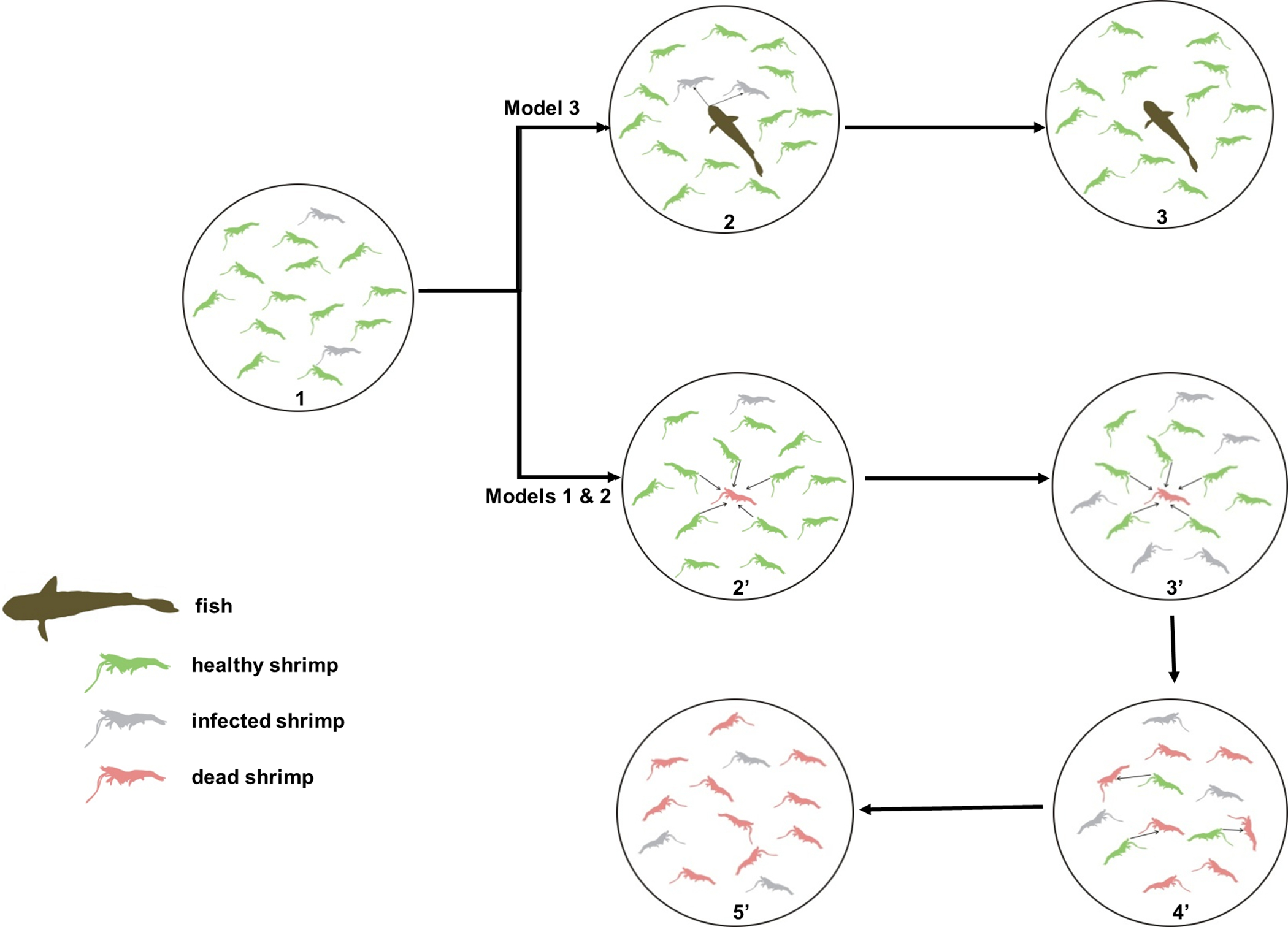
Spatiotemporal reconstruction of a predator–prey interface for biocontrol of pandemic viral diseases in shrimp aquaculture. The established model is based on the cycle of a dynamic process from healthy shrimp feeding on white spot syndrome virus (WSSV)-infected dead shrimp and converted to infected shrimp and to dead shrimp in a closed ecosystem (pond). The dynamic changes of three states (healthy, infected, and dead shrimp) in cultured shrimp influence the WSS (white spot syndrome) epidemic. Model 1 and Model 2: no fish (predator). Model 3: with fish (predator).

Specifically, we proposed a solution. “Polyculture in aquaculture, which means cultivating more than one species in the same pond, might maximize yield and reduce wastes in effluent through better utilization of the available food in the system. Therefore, polyculture has been considered a promising strategy in the future sustainable shrimp aquaculture industry. As the general theory predicts that selective predation on infected individuals can reduce the prevalence of diseases in the prey population, polyculture might prevent WSSV outbreaks by restoring the spatiotemporal interaction of predators and prey (Figure 1). Here, we developed a cost-effective and convenient shrimp polyculture system that effectively prevents outbreaks of WSS by introducing specific fish. The system is highly robust and has been demonstrated to successfully control WSS outbreaks in the cultivation of major cultivated marine shrimp species, including Pacific white shrimp (Litopenaeus vannamei), black tiger shrimp (Penaeus monodon), kuruma shrimp (Marsupenaeus japonica), and Chinese white shrimp (Fenneropenaeus chinensis). The implementation of this polyculture system does not require taking biosecurity measures.

Cycle one: 1 ◇2’◇3’: Fish root out diseased and dead shrimp.

1. Virus-carrier shrimp are manifested to be diseased shrimp upon environmental stress, initiating the WSS transmission in a shrimp population.
2. Fish prey on diseased shrimp and dead shrimp.
3. Fish root out diseased and dead shrimp, keeping the shrimp population healthy in the pond.

Cycle two: 1◊2◊3◊4◊5: WSS transmission process through healthy shrimp preying on dead shrimp.

1. Virus-carrier shrimp are manifested as diseased shrimp upon environmental stress, initiating the WSS transmission in the shrimp population.
2. Diseased shrimp become dead, and healthy shrimp prey on dead shrimp.
3. These predator shrimp become diseased shrimp. Other healthy shrimp continue to prey on dead shrimp.
4. These predator shrimp go straight to prey on dead shrimp. The infection cycle (from #2 to #4) continues in the shrimp population in a pond.
5. The infection cycle drives the WSS outbreak, leading to a shrimp population collapsing in a pond.

Furthermore, the system can control WSS outbreaks even when there are WSSV carriers in shrimp post larvae. Thus, small-scale farms can easily adopt this system to prevent WSS outbreaks without additional investment (Wang et al., 2021).

“After the promotion of the polyculture system in 2015, the farmers cultivated 8000/ha of shrimp in the ponds, which substantially increased the yield of shrimp from 175 ± 19 kg/ha to 1159 ± 135 kg/ha,” by which the polyculture system alleviates the poverty of small-scale farmers (Wang et al., 2021).

Our team also modelled ways to create a tunable, generalizable, and adoptable tool-based ecosystem to improve the environmental performance of blue food shrimp production. The system is easy to implement and requires no special biosecurity measures. The promotion of this system in China demonstrated that it allowed small-scale farmers to improve their livelihood through shrimp cultivation by controlling WSS outbreaks and increasing the production of ponds.

## 3 The challenges and the opportunities in restoring the diversity of the world’s aquatic “blue” food systems

Extrapolating the principle out of this study and putting it into practice might help “harness the world’s aquatic ‘blue’ food systems to help end hunger.” The retrospective literature comes in different perspectives, viewing the same predator–prey relationship as follows.

Nature published (August 25, 2016) an article on fostering synergies between biodiversity conservation and high multifunctionality levels, potentially promoting high levels of the multiple ecosystem services upon which human well-being depends (Soliveres et al., 2016).“Such functional importance of biodiversity in real-world ecosystems has been greatly underestimated due to focusing on individual trophic groups.” They focused on how species richness and abundance changes across multiple trophic groups affect the provision of multiple ecosystem services in real-world grassland systems. Their main conclusion is that diversity at multiple trophic levels is essential in maintaining ecosystem multifunctionality. In the opposite direction, changes in the biodiversity of an ecosystem can affect ecosystem multifunctionality. For example, if components of biodiversity are lost, the overall state and functioning of the system can be impaired (Scherber et al., 2006)—leading to a loss of ecosystem multifunctionality. “Such effects of species loss are predicted to be affected by both the number of species lost within a trophic level (horizontal diversity) and the number of trophic levels lost (vertical diversity) (Srivastava and Bell, 2009)” with subsequent failure in production, a problem that exists in intensified modern farming. While current research focuses on multiple ecosystem services in natural world grassland systems (Scherber et al., 2010), we applied this principle for concentrating on the effect of the presence/absence of a single predator species on a single prey species, a gap that has not been filled in seafood production of economic significance.

In our 30-year laboratory and field experiments, we realized that single-species intensive farming reduces the ecosystem’s biodiversity, and diminishing biodiversity is closely related to the occurrence of epidemic disease (Wang et al., 2021). The global pandemic of white spot syndrome (WSS), caused by the white spot syndrome virus (WSSV), bears a devastating loss in farmed shrimp production. Currently, there is no effective control for WSS. Through 30 years of field research, we found that the WSS pandemic occurs depending on WSSV transmission by which healthy shrimp consume WSSV-infected shrimp—the root cause of the WSS outbreak in the shrimp-farming ecosystem. This is due to the lack of predators consuming the WSSV-infected shrimp to cut off WSSV transmission in conventional single-species intensive shrimp production (Figure 1). After surveying biodiversity in naturally functioning ecosystems, we introduce a driving force of interaction between predator (fish) and prey (shrimp), thereby establishing biodiversity in farmed shrimp ponds to promote a functioning ecosystem in controlling WSS for biomass homeostasis balance (Figure 1). We determined releasing 1-kg grass carps (Ctenopharyngodon idellus) (predator) after evaluating 18 species of predictor fish for their capacity to cut off WSSV transmission. We scaled up for shrimp (Litopenaeus vannamei) farm production, successfully controlling WSS and producing an average yield (6375–9428 kg/hm2) in 105.53 hectares (hm2) of 320 ponds compared with an average yield (500 kg/hm2) in control ponds of a similar scale. Thus, we developed a novel biodiversity-based aquaculture ecosystem functioning to control a viral disease, suggesting a new way for seafood security.

The naturally occurring biodiversity (Chapin et al., 1997) helps sustain food webs (Chapin et al., 2000) and controls certain epidemic diseases (Crowder et al., 2010), leading to increased ecosystem productivity (Tilman et al., 2006). The loss of biodiversity is happening everywhere, and it is more evident in agricultural production, especially in intensive single-species output (Chapin et al., 2000). Intensive aquaculture is mainly the characteristic of single species (Tilman et al., 2006), high-density per unit area, and high yield (Crowder et al., 2010). However, intensive aquaculture reduces the biodiversity of an ecosystem (de Mazancourt et al., 2013), (Loreau et al., 2001) and results in epidemic diseases and economic losses (Altieri, 2004) (Crowder et al., 2010), especially it is more prominent in invertebrates because of a lack of adaptive immunity (SÁCHEZ-PAZ, 2010); thus, vaccination is not useful in disease control (Musthaq and Kwang, 2011), which leads to large-scale use of chemical substances. In 2011, China’s chemical drugs used in aquaculture amounted to 1.3 billion Chinese yuan (American dollars~$210 million). Chemical drug use causes environmental problems. Even worse, for some diseases, in particular for viral illnesses, these chemicals are not effective (Tendencia and de la Peña, 2001; Le et al., 2005; Lakshmi et al., 2013).

## 4 The theoretical framework of enclosed ecosystems and open ecosystems

Complementary to the Nature publication (Soliveres et al., 2016), our research restores biodiversity to drive the ecosystem functioning in aquaculture by engineering a single predator–prey (well-defined target species, one prey, paired with one predator) with a tightly regulated density in an enclosed system (pond, well-constructed ecosystem) for high yield production. In such an enclosed system, we can effectively avoid three detrimental factors in open land systems as described by Lester and Harmsen, (2002), such as 1) predator–prey migration rate and density change; 2) asymmetric growth rates between prey and predator; and 3) inference between predators. Critically evaluating by weighing in on those three factors leads to a conclusion—in a closed system, we can create a targeted, effective, sustainable, and stable interaction between predator and prey populations to successfully control a viral disease. To our knowledge, this is the first report on the control of aquatic viral diseases with a predator to restore ecosystem functioning.

Our research uses biodiversity and develops a new application of the predator–prey interaction in aquaculture. Ecologists have developed theories and models to capture the essence of the prey–predator relationship (Table 1). In nature, the interaction between predators and prey is dynamic, non-specific, and has a time-lag effect. Both predator and prey populations are not constant and not sustainable due to asymmetric growth rates between prey and predator. A non-specific prey–predator relationship significantly reduces the effectiveness of a biological control system. On the other hand, even with the predator–prey specificity, increasing a prey population leads to increasing its predator population, which in turn leads to the decline of the prey population, thereby reducing the predator population. In an open ecosystem, the complementary effects of predators on the suppression of prey depend on how effectively predators respond to variation in encounter rates and the quality of prey. Thus, a predator population may not completely control a prey population in farm production, leading to a low production yield (Hogg et al., 2013). In a closed ecological system, however, we established the sustainable stability of the predator–prey population interaction for the control of shrimp WSS, achieving a high production yield.

Such effectiveness of a predator–prey pairwise system has been discussed previously. Ives et al. (2005) introduced two concepts: biodiversity and ecosystem functioning (BEF) and multispecies predator–prey interactions (PPIs). Both BEF and PPI address the additive effects of diversity of single trophic-level systems on resource partitioning in predator–prey interactions. This consumer–resource model predicts the indirect and non-additive interactions in the densities of both resources (prey) and consumer (predator) species. They found that predator consumer diversity could have top–down effects on prey resources, while prey resource diversity could have bottom–up effects on predator consumers. Lester and Harmsen, (2002), however, found that functional and numerical responses do not always indicate the most effective predator for biological control based on their analysis of two predators in a two-prey system. They pointed out that functional and numerical assays on single predator–single prey systems in simplified laboratory environments do not allow predictions of the growth of mixed populations in realistic habitats or of the effectiveness of predators as biological control agents in the field due to the complex nature of predator–prey interactions. The problem with their study is that the polyphagous nature of predators allows them to attack non-target or alternative prey. Such a complex multi-level predator–prey relationship contributes little to the control of the target species (Louda et al., 1997). In an open ecosystem, there are issues with predator and prey emigration and immigration; therefore, the use of predator for prey is difficult to control. For example, the discrepant efficacies in large-scale open field applications of Trichogramma spp. egg parasitoid wasps rearing in vitro (Li and Gao, 1987) (predator) of pests (prey) in agriculture and forests in China (Gao et al., 1982; Cao et al., 1988) for 3 decades (Lu et al., 2017) have prompted us to realize that biological control works best in an enclosed ecosystem.

Here, we engineered a single predator–prey (well-defined target species, one prey, paired with one predator) with a tightly regulated density in an enclosed system (pond, well-constructed ecosystem) as supervised by a mathematically modeled artificial ecosystem (Figure 1). In such an enclosed system, we can effectively avoid three detrimental factors as described by Lester and Harmsen, namely, 1) predator–prey migration rate and density change; 2) asymmetric growth rates between prey and predator; and 3) inference between predators. Critically evaluating by weighing in on those three factors leads to the conclusion—in a closed system, we can create a targeted, effective, sustainable, and stable interaction between predator and prey populations for the successful control of a viral disease. To our knowledge, this is the first report on the control of aquatic viral disease with a predator.

Our artificial ecosystem is grounded on the wisdom by Buddha philosophers in ancient China (1252–1364; Song dynasty) of describing the biological balance of nature as XS-XK (相生-相克, 五行金木; 宋·释普济I五灯会元J卷四十六). The XS-XK (相生-相克: pronounced Xiāng Shēng-Xiāng Kè, abbreviated as XS-XK) relationship is indicated as intertwined as two sides of one coin: XS (相生) for mutual survival (positive regulation, yang) and XK (相克) for mutual restraint (negative regulation, yin). In conventional intensive aquaculture systems, human provides the necessary conditions (artificial nutrients and supplemental oxygen, etc.) to farm a single population for maximizing productivity; however, this breaks XS-XK’s balance because it lacks XK, leading to WSS occurrence. We introduce an XK fish competing with healthy shrimp for eating dead shrimp, leading to cutting off the WSSV route of transmission.

The effectiveness of XS-XK balance in governing natural ecosystems depends on biodiversity. However, modern agriculture uses chemical pesticides, resulting in reduced ecosystem biodiversity, all violating the XS-XK principle. Traditional and organic agriculture is dependent on biological prevention and control, preserving biodiversity and leading to an XS-XK-mediated ecosystem structure harmony (Steudel et al., 2012), (Macfadyen et al., 2009). They have defined the main elements of a functional ecosystem (Matson et al., 1997). These include maintaining biodiversity (Chapin et al., 2000), species richness (Silvertown et al., 1999; Wittebolle et al., 2009) and evenness (Hillebrand et al., 2008; Turnbull et al., 2012), abundance of species (Silvertown et al., 1999), niche partitioning (Finke and Snyder, 2008), and food webs (7, 31). Integrating these elements in modern agriculture production may restore the functional ecosystem (Benayas et al., 2009) such as rice–fish systems (Frei and Becker, 2005). All of these practices succeed to a certain extent but suffer in productivity.

## 5 Perspectives

Our pairwise prey–predator (shrimp–fish) system establishes an efficient XS-XK dynamics. We construct a shrimp–fish ecosystem, fish feeding a small amount of healthy shrimp, if no WSS occurs, leads to some loss of farm production (XK function); however, in case of a WSS outbreak, fish feeding large numbers of dead and diseased shrimp cuts off WSSV transmission, prevents the spread of WSS, and plays a positive role in farm production (XS function). To balance the XS-XK ecosystem and improve shrimp production, this study provides a solution about the type and quantity and body weight of fish in control of WSS outbreak.

In an aquaculture ecosystem, building XK, an ecological niche, needs to meet three criteria: necessity, targeting, and efficiency. Necessity is to introduce an XK niche (constraint niche) by using a restrained species. Targeting addresses the specificity—a predator specifically controls prey. Management of necessity and targeting will guarantee the efficiency—a predator completely controls prey. In 2014, the controlling WSS shrimp–fish system was widely applied in Chinese cultured shrimp, with an application area of about 100,000 hectares, resulting in the incidence of WSS in shrimp production dropped to below 5%. Whether or not crop farming can take advantage of the XK-XS niche to establish an efficient ecosystem, harmonization of modern and traditional farming is worth exploring.

## Figures and Tables

**Table 1.** Prey-predator or host-parasitoid interaction dynamics shape ecosystems and can help maintain biodiversity, which can be adopted for population dynamics modeling of biological control

Prey-predator	Ecosystem	Effectiveness of biocontrol (EBC)	Citation

Cotton aphid - ladybird beetles	Cotton field	Efficacy 50%	(Zhang et al., 2022)
Daphnia magna - larval dragonflies *Aeshna juncea*	Water	Effective ratio (3:1)	(Hirvonen and Ranta, 1996)
Copepods - mosquito larvae (malaria vector)	Water	Effective ratio (10:1)	(Pernia et al., 2007)
Invertebrate - vertebrate	Water	Effective ratio	(Miller-Ter Kuile et al., 2022)
Bacterium *Vibrio splendidus* - marine deposit feeder sea cucumber Apostichopus japonicus	Sea water	Partial role reversal	(Kaitala et al., 2021)
			
Lizard - snake	Island	Behavior	(Landry Yuan et al., 2021)
Fish - Marine mammal species (dolphins, whales, porpoises and seals)	Water	Observation	(Spitz et al., 2014)
Phytophagous insects - birds	Oilseed rape fields	Behavior	(Seree et al., 2021)
Prey - predator spatial physical habitat selection of joint spatial patterns of eight mobile marine species (grey seal, harbor seal, harbor porpoise, common guillemot, black-legged kittiwake, northern gannet, herring, and sandeels)	Sea water	Behavior	(Sadykova et al., 2017)
Four major trophic guilds (piscivores, invertivores, planktivores, and herbivores)	Tropical reefs	Predator-prey mass ratio	(Coghlan et al., 2022)
Small rodents (*Arvicola terrestris* voles) - red foxes	Land	Surveillance	(Umhang et al., 2021)
Prey-predator or host-parasitoid relationships	Land fields	Economic threshold modeling on a spatiotemporal scale	(Lima et al., 2009)
13 *Vibrio vulnificus* strains of different genotypes isolated from diverse environments were exposed to predation by the ciliated protozoan Tetrahymena pyriformis (Only strain ENV1 was resistant to predation).	Laboratory	bacteria evolve antipredator mechanisms: changing morphology, biofilm formation, and secretion of toxins or virulence factors.	(Rasheedkhan Regina et al., 2022)
Mus musculus by *Toxocara canis* or *Toxoplasma gondii* - *Mus musculus* (Balb/c)	Laboratory	Mice chronically infected by *Toxoplasma gondii* showed impaired learning and short-term memory	(Correa et al., 2014)
Scorpion *Bothriurus bonariensis* (Bothriuridae) to prey on harvestmen (*Acanthopachylus aculeatus*, *Discocyrtus prospicuus*, *Parampheres bimaculatus* and P*achyloides thorellii* (Gonyleptidae)).	Land	Scorpions control harvestmen	(Albin and Toscano-Gadea, 2015)
sharp tooth African catfish *Clarias gariepinus* (Burchell 1822) on all other fish species	tropical reservoir	Adverse effects of African catfish on all fish	(Khan et al., 2021)

Note: Refer to below references for details.
